# The "Red Cross" Light Cure and Electrical Institute

**Published:** 1904-03-12

**Authors:** 


### THE "RED-CROSS" LIGHT CURE AND ELECTRICAL INSTITUTE.

The increasing acceptance among medical men and the public of light and electric treatment for various diseases, accounts for the opening of an institute such as this, which is situated at 35 Edgware Road (near the Marble Arch). Very few medical men have such accommodation as would permit of their setting up an installation for these forms of treatment, and, indeed, in very few private practices would it be profitable to do so. For a practitioner it is more convenient often to have a patient for whom he thinks this treatment desirable receive it under his own supervision, and according to his own prescription, at such an institute as this. As, however, there may be patients from the country whose medical attendants cannot accompany them, it has been arranged that a qualified medical man can be consulted at the institute itself. Examinations are made by the Roentgen rays, and there are both male and female qualified nurses to carry out the treatment recommended. The apparatus includes light and electric baths of various kinds. One of these, a circular enclosure, starred with incandescent electric lights, takes the place of the ordinary Turkish bath, with the advantage that the patient sits with his head above the bath, breathing ordinary air, and so is not so liable to the feeling of faintness which is to so many people a reason for avoiding this kind of bath. Another bath, otherwise similar, has the incandescent light supplemented by the arc-light, which shines through blue-glass slides, and is meant for the treatment of local ailments. There is also a douche bath where the electricity is passed through the water and can be localised at the will of the operator on any desired part. Cradle apparatus of different sizes, lined with rows of light, are provided for local affections, so that the treatment can be applied to one limb only in suitable cases. There is also a light installation for the treatment of skin diseases, and comfortable rooms where patients can rest after the séance.

